# Intra-individual dynamic comparison of ^18^F-PSMA-11 and ^68^Ga-PSMA-11 in LNCaP xenograft bearing mice

**DOI:** 10.1038/s41598-020-78273-7

**Published:** 2020-12-03

**Authors:** Sarah Piron, Jeroen Verhoeven, Benedicte Descamps, Ken Kersemans, Kathia De Man, Nick Van Laeken, Leen Pieters, Anne Vral, Christian Vanhove, Filip De Vos

**Affiliations:** 1 Laboratory for Radiopharmacy, Ghent University, Ottergemsesteenweg 460, 9000 Ghent, Belgium; 2 IBiTech-MEDISIP, Department of Electronics and Information Systems, Ghent University, Ghent, Belgium; 3 Department of Medical Imaging, Ghent University Hospital, Ghent, Belgium; 4 Department of Human Structure and Repair, Ghent University, Ghent, Belgium

**Keywords:** Urological cancer, Diagnostics, Preclinical research, Cancer imaging

## Abstract

Recently, a ^18^F-labeled derivative of the widely used ^68^Ga-PSMA-11 was developed for PET imaging of prostate cancer. Although ^18^F-PSMA-11 has already been evaluated in a Phase I and Phase II clinical trial, preclinical evaluation of this radiotracer is important for further understanding its dynamic behavior. Saturation binding experiments were conducted by incubation of LNCaP cells with ^18^F-PSMA-11 or ^68^Ga-PSMA-11 for 1 h, followed by determination of the specific and aspecific binding. Mice bearing LNCaP or PC-3 xenografts each received ± 3.7 MBq ^18^F-PSMA-11 and ^68^Ga-PSMA-11 followed by dynamic acquisition of 2.5 h as well as ± 15 MBq ^18^F-FDG followed by static acquisition at 1 h post injection (p.i.). Uptake was evaluated by comparison of uptake parameters (SUV_mean_, SUV_max_, TBR_mean_ and TBR_max_). Mice underwent ex vivo biodistribution where ^18^F-PSMA-11 activity was measures in excretory organs (kidneys, bladder and liver) as well as bone fragments (femur, humerus, sternum and skull) to evaluate bone uptake. The dissociation constant (K_d_) of ^18^F-PSMA-11 and ^68^Ga-PSMA-11 was 2.95 ± 0.87 nM and 0.49 ± 0.20 nM, respectively. Uptake parameters were significantly higher in LNCaP compared to PC-3 xenografts for both ^18^F-PSMA-11 and ^68^Ga-PSMA-11, while no difference was found for ^18^F-FDG uptake (except for SUV_max_). Tumor uptake of ^18^F-PSMA-11 showed a similar trend over time as ^68^Ga-PSMA-11, although all uptake parameter curves of the latter were considerably lower. When comparing early (60 min p.i.) to delayed (150 min p.i.) imaging for both radiotracers individually, TBR_mean_ and TBR_max_ were significantly higher at the later timepoint, as well as the SUV_max_ of ^68^Ga-PSMA-11. The highest %ID/g was determined in the kidneys (94.0 ± 13.6%ID/g 1 h p.i.) and the bladder (6.48 ± 2.18%ID/g 1 h p.i.). No significant increase in bone uptake was seen between 1 and 2 h p.i. Both radiotracers showed high affinity for the PSMA receptor. Over time, all uptake parameters were higher for ^18^F-PSMA-11 compared to ^68^Ga-PSMA-11. Delayed imaging with the latter may improve tumor visualization, while no additional benefits could be found for late ^18^F-PSMA-11 imaging. Ex vivo biodistribution demonstrated fast renal clearance of ^18^F-PSMA-11 as well as no significant increase in bone uptake.

## Introduction

Prostate specific membrane antigen (PSMA) is a transmembrane glycoprotein with glutamate carboxypeptidase activity. It is an excellent target for specific imaging as well as targeted therapy in almost all subtypes of prostate cancer due to overexpression, which is enhanced in poorly differentiated, metastatic and hormone-refractory disease^1^. Out of the extensive pool of PSMA targeting PET probes that have already been developed, ^68^Ga-PSMA-11 is the most widely studied and used radiotracer in clinical practice. A recent meta-analysis of 29 studies by Hope et al*.*^2^, focusing on histopathological validation, reported a sensitivity and specificity of 0.74 (95% CI, 0.51–0.89) and 0.96 (95% CI, 0.84–0.99), respectively, at initial staging. At biochemical recurrence (BCR), good detection rates were achieved for both PSA values above 2.0 ng/mL (0.94; 95% CI, 0.91–0.96) and below 2.0 ng/mL (0.63; 95% CI, 0.55–0.70), demonstrating the possibility of early detection of BCR in patients with low PSA values. These results are similar to findings of Eiber et al*.*^3^ who reported detection rates of 96.8% for PSA values ≥ 2.0 ng/mL and 93.0%, 72.7% and 57.9% for PSA values of 1 to < 2 ng/mL, 0.5 to < 1 ng/mL and 0.2 to < 0.5 ng/mL, respectively.

Despite the high affinity for the PSMA receptor and the excellent results with regard to currently used PET probes^4–7^, the use of ^68^Ga as radionuclide is associated with some unfavorable physical properties. In comparison to ^18^F, ^68^Ga has a shorter half-life (68 min vs 110 min), as well as a lower positron emission (89% vs 97%) and a higher maximum positron energy (1.90 meV vs 0.63 meV), resulting in a longer positron range and lower spatial resolution^8^. Furthermore, the cyclotron-based production of ^18^F makes large batch production possible as opposed to the limited capacity of 2–3 patient doses for the generator-produced ^68^Ga^9^. Amongst others, the well-established use of ^68^Ga-PSMA-11 has led to the development of the fluorine-18 derivative ^18^F-PSMA-11 by Malik et al*.*^10^ and Boschi et al*.*^11^ and was further optimized by Kersemans et al*.*^12^ to enable semi-automated production. The Phase I clinical trial conducted in our hospital evaluated safety, dosimetry and biodistribution^13^. The recently published Phase II study reported on an optimized scan protocol where dosage, scan time and administration of a diuretic were studied^14^.

Although the use of ^18^F-PSMA-11 has already been investigated in 107 patients, preclinical evaluation of this radiotracer is warranted in order to gain a deeper understanding of its dynamic character, biological behavior and excretion kinetics. Therefore, imaging characteristics of ^18^F-PSMA-11 and ^68^Ga-PSMA-11 were compared in a preclinical setting. To our knowledge, no dynamicly acquired intra-individual comparison of these two radiotracers as well as extensive in vivo and ex vivo evaluation of bone uptake of ^18^F-PSMA-11 tracer has been published before.

## Materials and methods

### Synthesis of PET radiotracers

Synthesis of ^18^F-PSMA-11 was performed as described by Kersemans et al*.*^12^ on a modified SynthraFCHOL synthesis module (Synthra GmbH, Hamburg, Germany). ^68^Ga-PSMA-11 was prepared using a lyophilized sterile cold kit (ANMI, Liege, Belgium) by reconstitution of 25 µg PSMA-11 precursor in acetate buffer (pH 4.1–4.4). ^68^Ga was eluted from a ^68^Ge/^68^Ga generator (50 mCi; IRE-Elit, Fleurus, Belgium) in an evacuated sterile vial using 1.1 mL of 0.1 M HCl and added to the precursor solution. Labeling was performed at room temperature for 5 min.

Radiochemical purity was determined by thin layer chromatography (TLC) using Alugram RP18-W/UV254 plates (Machery Nagel, Düren, Germany) and 3:1 (v/v) acetonitrile in water as mobile phase. To determine the specific activity (SA), high liquid performance chromatography (HPLC) was performed with a Prevail C18 reversed-phase column (4.6 × 250 mm, 5 µm, Lokeren, Belgium) at 40 °C and a mobile phase using a gradient system (Solvent A: water (0.1% TFA); Solvent B: acetonitrile; 0-4 min: 15% B, 4-11 min: from 15 to 70% B, 11-14 min: from 70 to 15% B and 14-16 min: 15% B) at a flow rate of 2 mL/min.

### Cell culture

Prostate carcinoma cell lines LNCaP (ATCC CRL-1740, PSMA positive) and PC-3 (ATCC CRL-1435, PSMA negative) were cultured using RPMI 1640 medium supplemented with 10% FBS, 1% streptomycine/penicillin (10,000 U/mL) and 1% glutamine 200 mM and maintained at 37 °C in 5% CO_2_ in humidified air.

### Affinity

Saturation binding experiments were conducted as described by Verhoeven et al*.*^15^ to determine the K_d_ of ^18^F-PSMA-11 and ^68^Ga-PSMA-11. Wells were seeded with 2 × 10^5^ LNCaP cells 48 h prior to the experiments using poly-lysine coated 24-well-plates (VWR, USA). After removal of the culture medium, wells were washed twice with 1 mL HEPES buffer (pH 7.4, 37 °C). Six dosing solutions between 2.5 and 50 nM of both radiotracers were prepared in HEPES buffer and evaluated in triplicate. Non-specific binding was determined by co-incubation with 100 µM 2-(phosphonomethyl)-pentanedioic acid (2-PMPA, Sigma Aldrich, Belgium). After an incubation period of 1 h at 37 °C, plates were cooled on ice and 1 mL ice-cold 1% BSA/PBS was added to stop radiotracer uptake. Cells were washed twice with 2 mL ice-cold PBS and subsequently lysed with 0.1 M NaOH (VWR, USA). ^18^F-PSMA-11 and ^68^Ga-PSMA-11 uptake in the cells was measured with an automated gamma counter (Cobra-inspector 5003, Canberra Packard, Meriden, CT, USA) and corrected for amount of protein by a Bicinchonic Acid (BCA) assay (ThermoFisher Scientific, Belgium). The K_d_ value was calculated by non-linear regression using Graphpad Prism 5.0 (GraphPad Software, San Diego, CA, USA, http://www.graphpad.com).

### Inoculation of mice

The study was approved by the Ghent University Ethical Committee on animal experiments (ECD 17/14). All animals (n = 10) were kept and handled according to the European guidelines (Directive 2010/63/EU) and housed under environmentally controlled conditions (12 h normal light/dark cycles, 20–24 °C and 40–70% relative humidity) with food and water ad libitum. On the day of the inoculation, LNCaP and PC-3 cells were washed twice with FBS-free RPMI 1640 medium and two cell suspensions of 5 × 10^6^ cells/100 µL were prepared and kept on ice until inoculation. Four-week-old male athymic nude mice (swiss nu/nu, Charles River Laboratory, France) were subcutaneously injected with 200 µL 1:1 cell:Matrigel suspension using precooled insulin syringes on either side of each mouse (LNCaP, n = 6; PC-3, n = 4) at shoulder height. Tumor growth was monitored weekly for 5–6 weeks until tumors reached a diameter between 5 and 10 mm.

### Biodistribution

Eight male athymic nude mice (swiss nu/nu, Charles River Laboratory, France) were subjected to ex vivo biodistribution. One additional mouse bearing LNCaP xenograft was added to evaluate tumor uptake. All mice received 1.95 ± 0.10 MBq ^18^F-PSMA-11 and were sacrificed at 1 h (n = 4 + 1) or 2 h (n = 4) post injection (p.i.). Excretory organs (kidneys, bladder and liver) and bone fragments (femur, humerus, sternum and skull) were removed, weighted and measured using a gamma counter.

### PET imaging

Solutions of 20 MBq/µg ^18^F-PSMA-11 and 1.5 MBq/µg ^68^Ga-PSMA-11 were prepared by adding the appropriate amount of a 0.1 µg/µL PSMA-11 stock solution to 6–10 MBq solution of each radiotracer. After intravenous injection of 4.03 ± 0.26 MBq ^18^F-PSMA-11 or 3.82 ± 0.20 MBq ^68^Ga-PSMA-11 in the tail vein, all mice underwent two dynamic PET scans for 2.5 h. For tumor confirmation of PSMA negative PC-3 tumors, ^18^F-FDG PET scans were performed. Mice were fasted at least 6 h before tracer administration. One hour after injection of 14.37 ± 3.77 MBq ^18^F-FDG, mice underwent a 30 min static ^18^F-FDG PET scan. Each mouse (n = 10) underwent two dynamic (^18^F-PSMA-11 or ^68^Ga-PSMA-11) and one static (^18^F-FDG) PET scan within 10 days, each time followed by a CT scan for co-registration. Dynamic PET images were acquired in list mode using a dedicated small animal PET scanner (FLEX Triumph II, Trifoil imaging, Northridge, CA) with a spatial resolution of 1.3 mm and an axial field-of-view (FOV) of 7.5 cm. All PET scans were reconstructed into a 200 × 200 × 128 matrix by a 3D Maximum Likelihood Expectation Maximization (MLEM) algorithm (LabPET Version 1.12.1, TriFoil Imaging, Northridge CA) using 50 iterations and a voxel size of 0.5 × 0.5 × 0.59675 mm. The dynamically acquired PET data were reconstructed into 30 time frames of 5 min as well as 6 × 5 min and 4 × 30 min.

### Image analysis

Images were analyzed using the Amide software^16^. After co-registration of PET and CT images, volumes of interest (VOIs) were drawn manually for delineation of the tumor, kidneys, bladder and bone fragments (spine, femur, sternum and humerus). A background region was drawn in the same transversal slice as tumor VOIs. The tracer uptake in each tumor VOI was calculated as mean and maximum standardized uptake value (SUV_mean_ and SUV_max_) according to Formula 1.

1 $$ SUV = \frac{{(Maximum)\; Activity \;VOI \left( {\frac{MBq}{{mL}}} \right)}}{Injected \;dose\; (MBq)} \times Body\;weight \;(g). $$

Besides SUV_mean_ and SUV_max_, tumor-to-background ratios (TBR_mean_ and TBR_max_) were determined. For non-tumor tissues, only SUV_mean_ was determined. Semi-quantitative analysis of tumor uptake was performed for every 5 min time frame and plotted at 5, 10, 15, 20, 25, 30, 60, 90, 120 and 150 min.

### Immunohistochemical evaluation

After the last scan, mice were sacrificed and tumors were collected for immunohistochemical (IHC) evaluation as described by Braeckman et al*.*^17^. Sections were either stained using Hematoxylin and Eosin or incubated with a primary PSMA antibody (1:400, 2 h, Abcam, ab133579) and counterstained using hematoxylin (Mayer). Sections were digitally scanned with a virtual scanning microscope (Olympus BX51, Olympus Belgium SA/NV, Berchem, Belgium) at high resolution (40 × magnification).

### Statistical analysis

All uptake parameters (SUV_mean_, SUV_max_, TBR_mean_ and TBR_max_) were expressed as mean ± SEM. Curves were constructed using GraphPad Prism 5.0 (GraphPad Software, San Diego, CA, USA, http://www.graphpad.com). The statistical analysis was performed in R^18^ using the Wilcoxon-signed Rank test for the cross-over intra-individual comparison of radiotracer uptake and the Mann–Whitney *U* test for comparison of uptake between PSMA positive and negative tumors. The significance level was set on p ≤ 0.05.

## Results

### Synthesis

^18^F-PSMA-11 and ^68^Ga-PSMA-11 were both obtained with a radiochemical purity of ≥ 95% by TLC analysis. The SA at the end of synthesis (EOS) was 104.8 ± 81.6 MBq/µg for ^18^F-PSMA-11 and 20.5 ± 10.6 for ^68^Ga-PSMA-11. The mean injected activity and SA at time of injection was 4.03 ± 0.26 MBq and 19.67 ± 7.66 MBq/µg for ^18^F-PSMA-11 and 3.82 ± 0.20 MBq and 1.48 ± 0.15 MBq/µg for ^68^Ga-PSMA-11. ^18^F-PSMA-11 for the biodistribution study was obtained with a radiochemical purity of > 99.9% and SA of 182.52 MBq/µg. The mean injected activity and SA at time of injection were 1.95 ± 0.10 MBq and 91.3 ± 29.8 MBq/µg, respectively.

### Affinity

The dissociation constant (K_d_) in LNCaP cells was determined to be 2.95 ± 0.87 nM [95% CI, 0.54–5.36] for ^18^F-PSMA-11 and 0.49 ± 0.20 nM [95% CI, 0.0053–0.98] for ^68^Ga-PSMA-11.

### Image analysis

**Figure 1:**
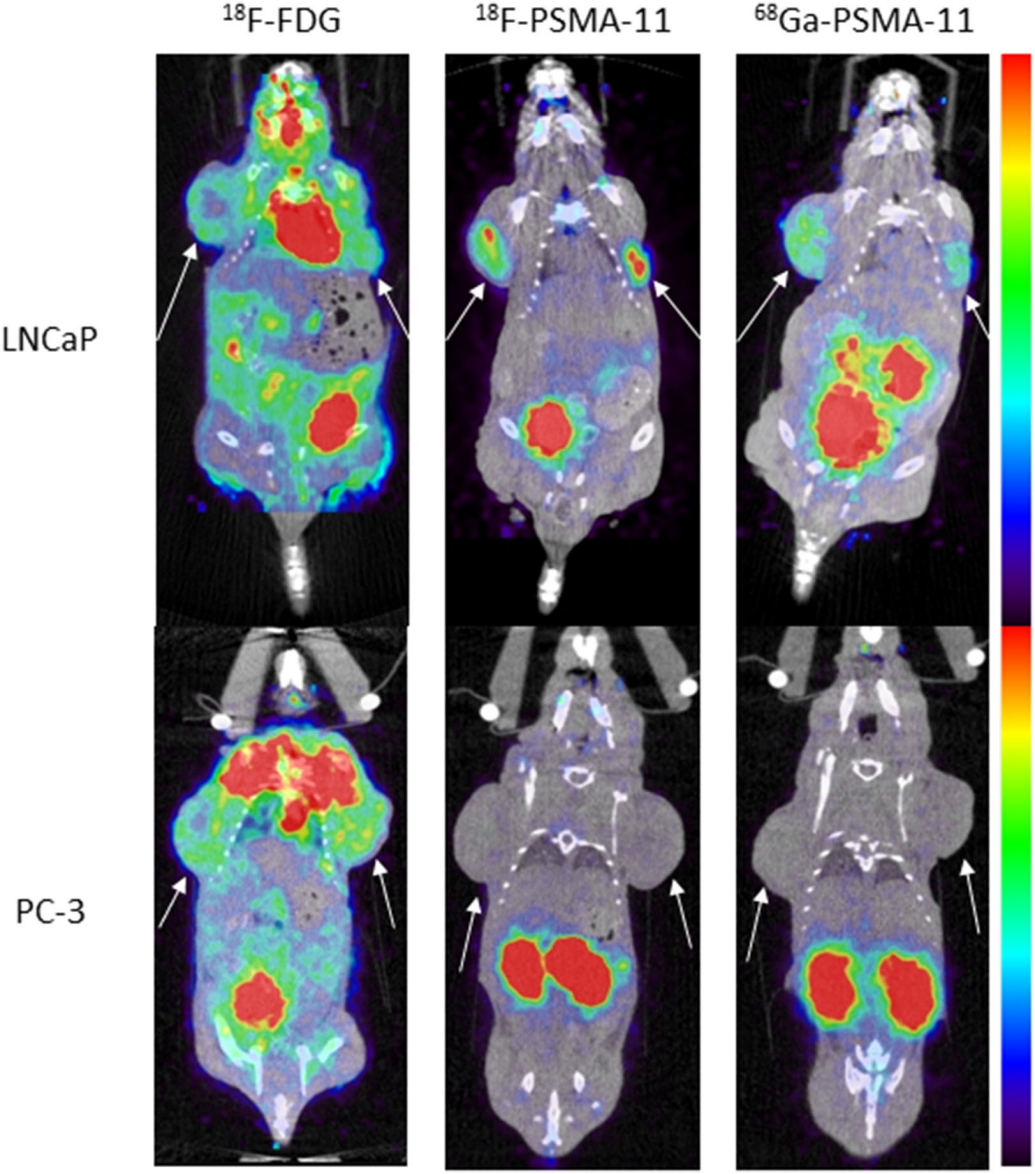
Comparison of ^18^F-FDG, ^18^F-PSMA-11 and ^68^Ga-PSMA-11 uptake in PSMA-positive (LNCaP) and PSMA-negative (PC-3) tumors (indicated by white arrows) 1 h p.i. Colormaps were adapted in order to optimally visualize the tumor.

**Figure 2:**
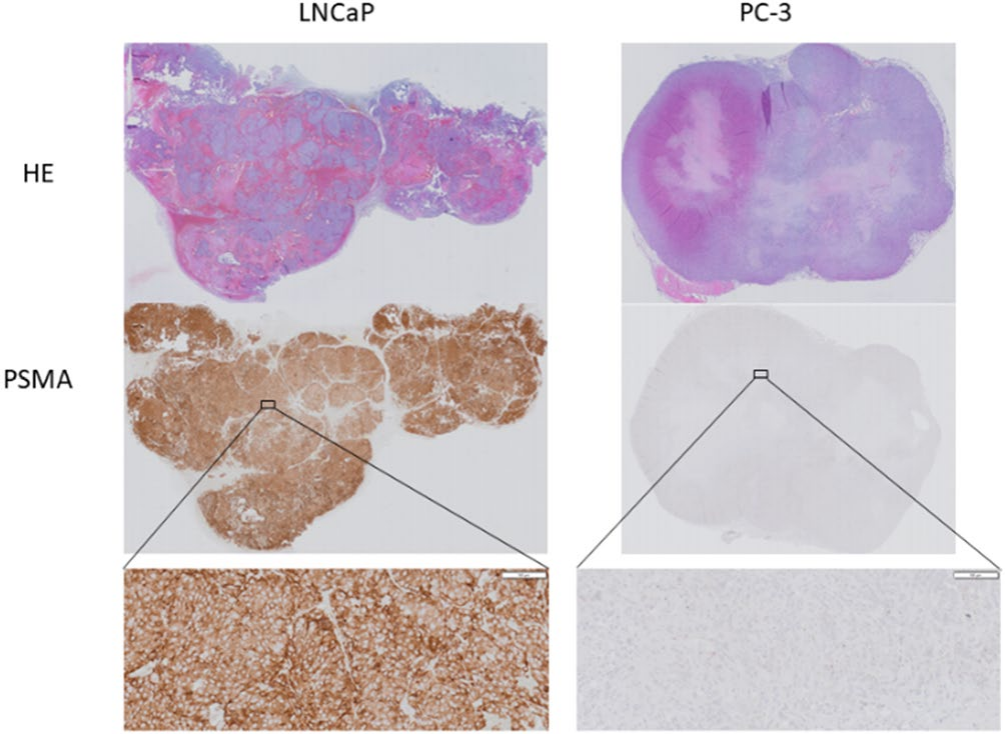
Immunohistochemical images of a representative PSMA-positive LNCaP tumor (left) and a PSMA-negative PC-3 tumor (right). Tumors are stained with Hematoxylin and Eosin (HE) and PSMA. Magnification × 40.

Each mouse underwent a dynamic ^18^F-PSMA-11 and ^68^Ga-PSMA PET/CT for 2.5 h and a static 30 min ^18^F-FDG PET/CT at 1 h p.i. within 10 days of each other. Representative images at 1 h p.i. of two mice with either PSMA positive (LNCaP) or PSMA negative (PC-3) xenografts are presented in Fig. 1. Colormaps were adapted in order to optimally visualize the tumor, images comparing radiotracers at identical thresholds can be found in the Supplementary Data (Figure S1). PSMA-targeting radiotracers showed less background activity in adjacent tissues compared to ^18^F-FDG. LNCaP tumors could be clearly identified with all three radiotracers, while PC-3 tumors were only visible with ^18^F-FDG. The specificity of ^18^F-PSMA-11 and ^68^Ga-PSMA-11 was visualized and semi-quantified by comparing radiotracer uptake in PSMA positive (LNCaP) and PSMA negative (PC-3) tumors. SUV_mean_, SUV_max_, TBR_mean_ and TBR_max_ were significantly higher in LNCaP compared to PC-3 xenografts for both ^18^F-PSMA-11 and ^68^Ga-PSMA-11, while no difference was found for these parameters with regard to ^18^F-FDG uptake, except for SUV_max_ (Table 1). The presence and absence of PSMA expression in respectively LNCaP and PC-3 cells was demonstrated with IHC analysis (Fig. 2).

**Table 1:** Uptake parameters SUV_mean_, SUV_max_, TBR_mean_ and TBR_max_ 60 min p.i. (T60) for all radiotracers (^18^F-PSMA-11, ^68^Ga-PSMA-11 and ^18^F-FDG) for LNCaP and PC-3 xenografts. Values are reported as mean ± SEM, p-values were calculated using the Mann–Whitney *U* test and corrected by Bonferroni for multiple testing.

T60	SUV_mean_			SUV_max_		
	LNCaP	PC3	p	LNCaP	PC3	p
^18^F-PSMA	2.59 ± 0.25	0.30 ± 0.03	< 0.001	5.59 ± 0.55	0.75 ± 0.06	< 0.001
^68^Ga-PSMA	0.98 ± 0.10	0.36 ± 0.03	< 0.001	3.27 ± 0.34	1.08 ± 0.08	< 0.001
^18^F-FDG	0.56 ± 0.09	0.72 ± 0.02	1	0.97 ± 0.13	1.52 ± 0.10	0.044

	TBR_mean_			TBR_max_		
	LNCaP	PC3	p	LNCaP	PC3	p
^18^F-PSMA	8.64 ± 1.06	1.62 ± 0.17	< 0.001	17.48 ± 2.26	4.63 ± 0.46	< 0.001
^68^Ga-PSMA	3.45 ± 0.56	0.97 ± 0.09	< 0.01	11.87 ± 2.18	3.38 ± 0.42	< 0.001
^18^F-FDG	1.60 ± 0.21	1.22 ± 0.08	1	2.82 ± 0.27	2.56 ± 0.24	1

**Figure 3:**
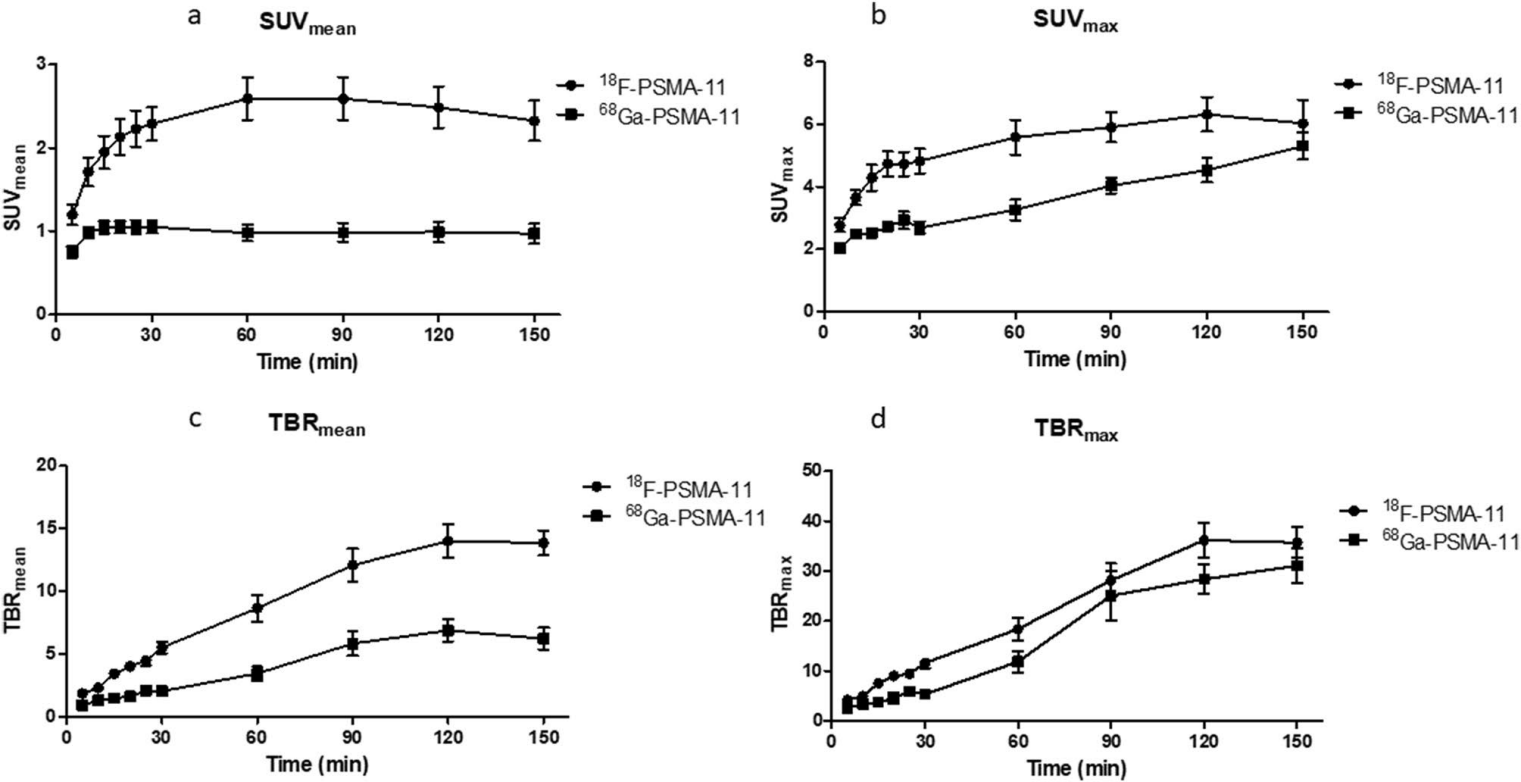
Comparison of ^18^F-PSMA-11 and ^68^Ga-PSMA-11 uptake in PSMA-positive (LNCaP) tumors regarding uptake parameters SUV_mean_ (**a**), SUV_max_ (**b**), TBR_mean_ (**c**) and TBR_max_ (**d**).

**Figure 4:**
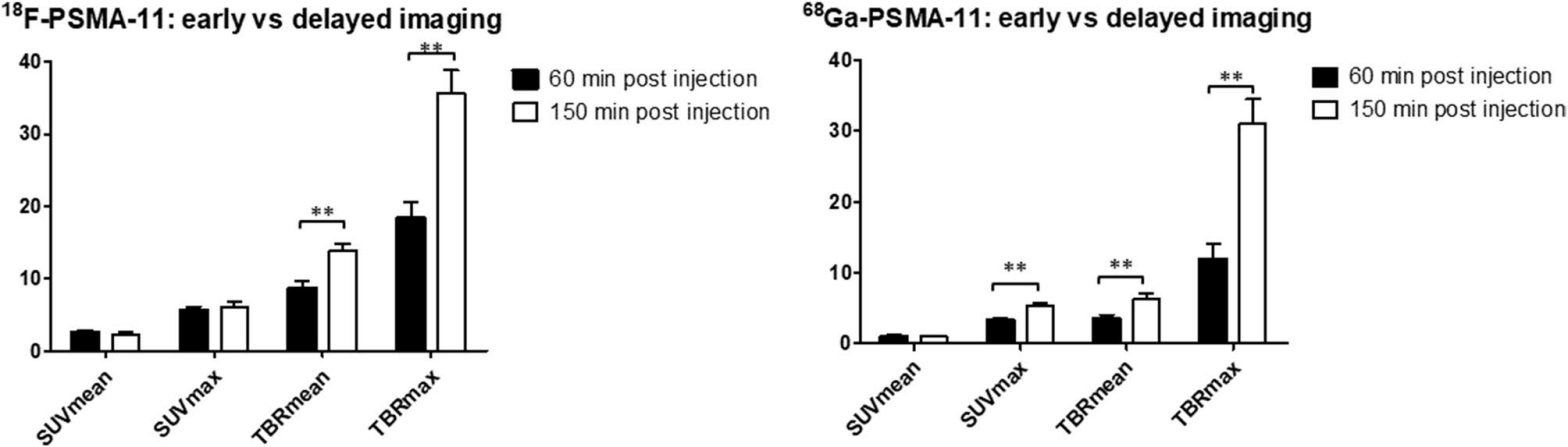
Comparison of early (60 min p.i.) and delayed (150 min p.i.) imaging of ^18^F-PSMA-11 and ^68^Ga-PSMA-11 in LNCaP tumors. *p < 0.05, **p < 0.01.

**Figure 5:**
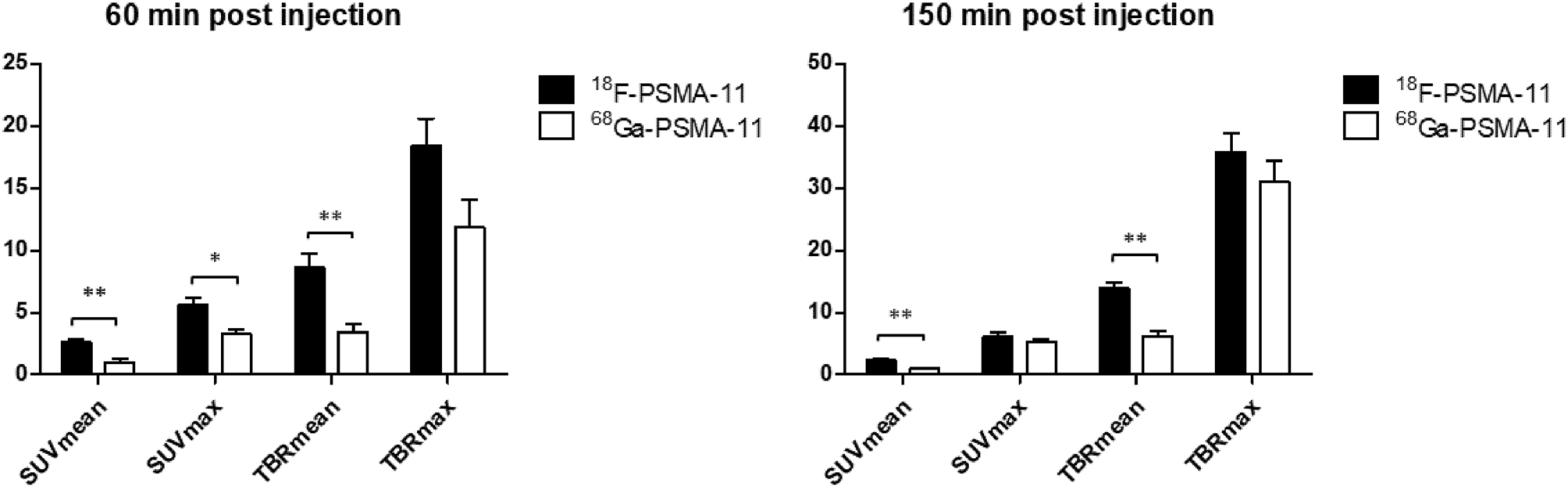
Comparison of tumor uptake in LNCaP tumors between ^18^F-PSMA-11 and ^68^Ga-PSMA-11 at 60 min and 150 min post injection. *p < 0.05, **p < 0.01.

Tumor uptake of ^18^F-PSMA-11 in LNCaP tumors increased rapidly within the first 30 min post radiotracer administration for all uptake parameters (Fig. 3). SUV_mean_ values reached a maximum between 60 and 90 min p.i. while SUV_max_ values increased up to 2 h p.i. TBR_mean_ and TBR_max_ values continued to increase up to 150 min p.i. Tumor uptake of ^68^Ga-PSMA-11 showed a similar trend over time, except for SUV_mean_ values, where no further increase could be seen after 20 min. When comparing early (60 min p.i.) to delayed (150 min p.i.) imaging for both radiotracers individually, TBR_mean_ and TBR_max_ were significantly higher at the later timepoint, whereas for ^68^Ga-PSMA-11 also an increased SUV_max_ was observed (Fig. 4). When comparing both radiotracers at 60 min and 150 min p.i., all uptake parameter values were higher for ^18^F-PSMA-11 compared to ^68^Ga-PSMA-11. These differences were significant, except for TBR_max_ and SUV_max_ 150 min p.i. (Fig. 5).

**Figure 6:**
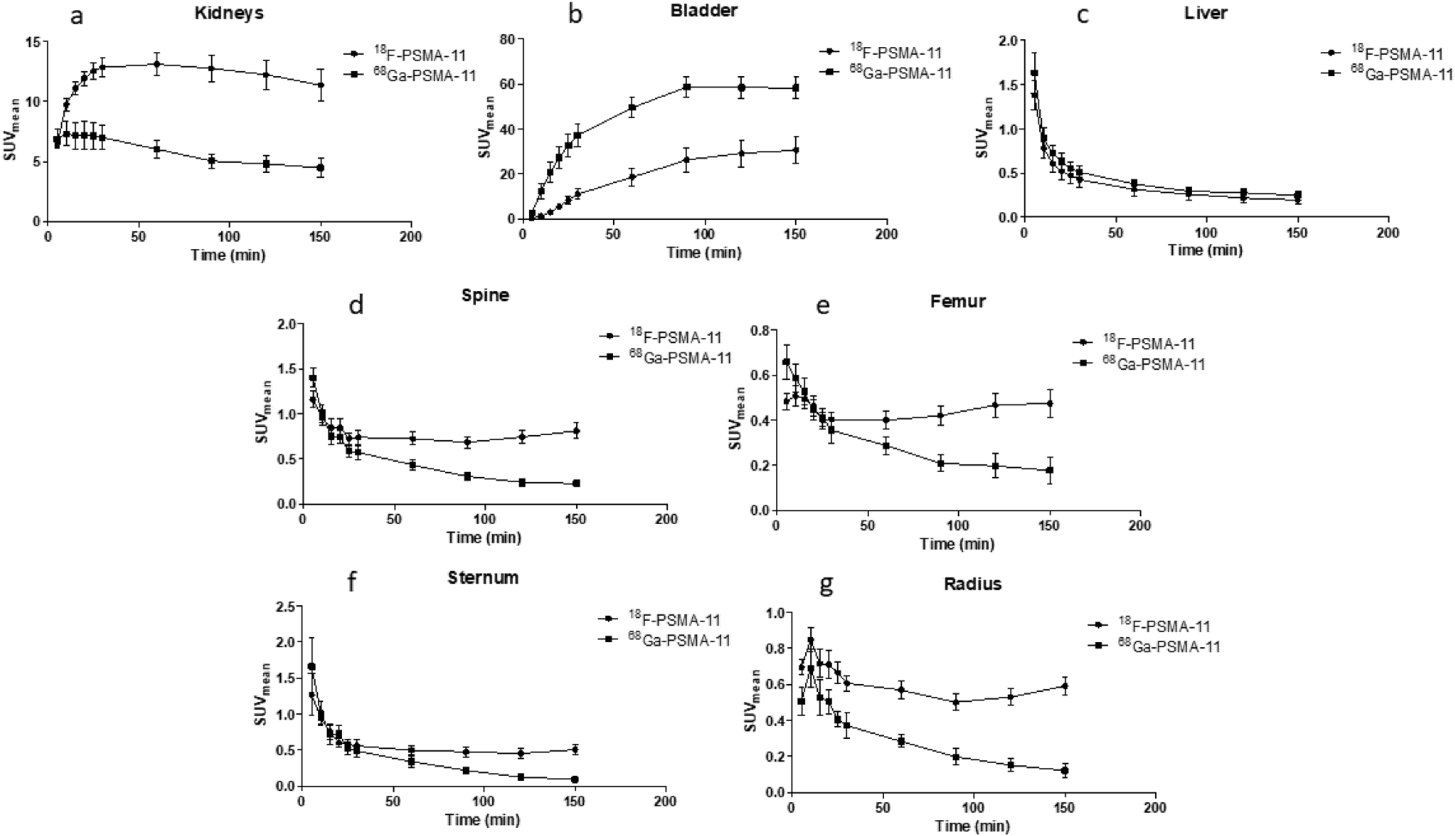
Time activity curves of ^18^F-PSMA-11 and ^68^Ga-PSMA-11 of excretory organs [kidney (**a**); bladder (**b**); liver (**c**)] and bone (spine (**d**); femur (**e**); sternum (**f**); humerus (**g**)].

**Figure 7:**
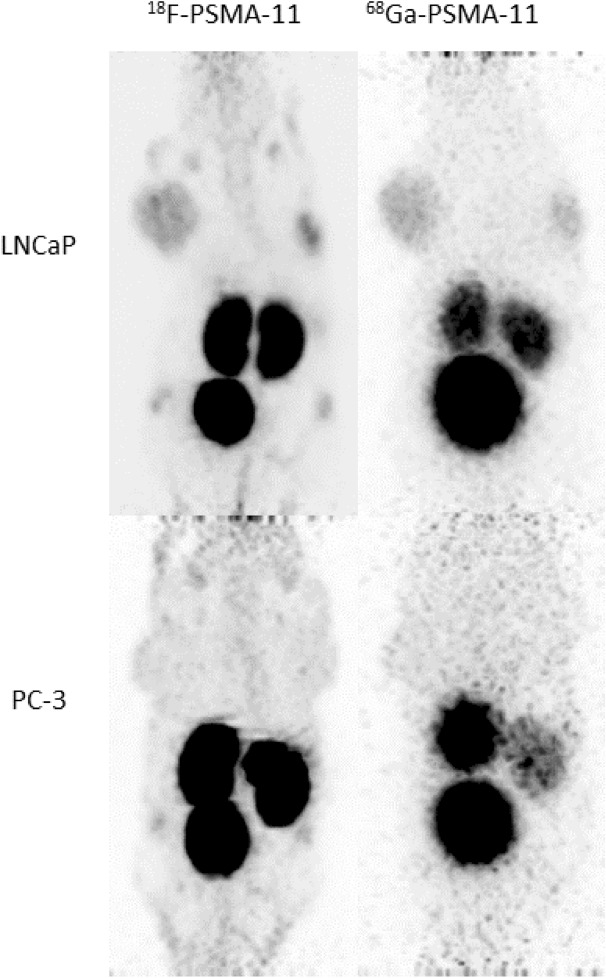
Maximum intensity projection (MIP) PET images at 1 h p.i. of two mice with either PSMA positive tumors (LNCaP, top) or PSMA negative tumors (PC3, bottom). Images show high kidney and bladder uptake and low/absent liver uptake, suggesting predominantly renal clearance.

Time activity curves of the excretory organs (kidneys, bladder and liver) demonstrated higher ^18^F-PSMA-11 radioactivity in the kidneys (SUV_mean_ 30 min p.i. of 12.98 ± 0.82 vs 7.20 ± 1.09) while ^68^Ga-PSMA-11 was more prominent in the bladder (SUV_mean_ 60 min p.i. of 49.71 ± 4.93 vs 16.82 ± 3.87) (Fig. 6), which was also visible on maximum intensity projection (MIP) images at 1 h p.i. (Fig. 7). Liver uptake decreased rapidly for both radiotracers indicating limited hepatobiliary clearance. Bone uptake was assessed using VOIs drawn in the spine, femur, sternum and humerus. The resulting SUV_mean_ of both ^18^F-PSMA-11 and ^68^Ga-PSMA-11 in these VOIs decreased during the first 60 min p.i. Between 60 and 150 min p.i., presence of ^68^Ga-PSMA-11 in the bone continued to decrease while the uptake of ^18^F-PSMA-11 slightly increased (SUV_mean_ from 0.71 ± 0.07 to 0.75 ± 0.07 in the spine (p = 0.359) and from 0.41 ± 0.04 to 0.47 ± 0.06 in the femur (p = 0.1851)) or remained constant (SUV_mean_ from 0.50 ± 0.07 to 0.51 ± 0.07 in the sternum (p = 0.4755) and from 0.57 ± 0.05 to 0.59 ± 0.05 in the humerus (p = 0.7598)).

### Biodistribution

**Figure 8:**
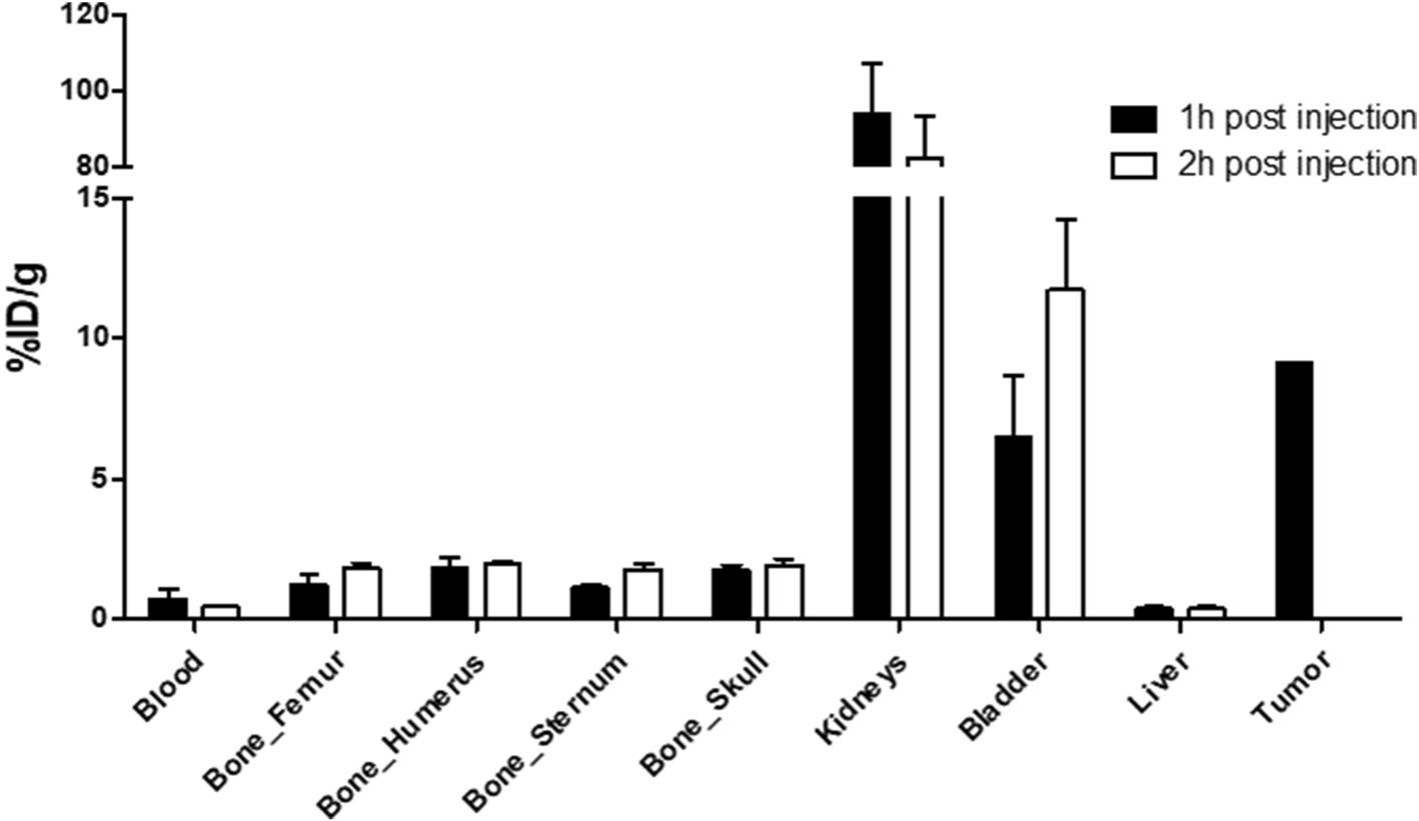
Visual presentation of ex vivo biodistribution 1 h and 2 h p.i. of ^18^F-PSMA-11.

Blood levels of ^18^F-PSMA-11 decreased between 1 and 2 h p.i. from 0.75 ± 0.31%ID/g to 0.47 ± 0.03%ID/g. The highest %ID/g was determined in the kidneys (94.0 ± 13.6%ID/g 1 h p.i. and 82.5 ± 10.9%ID/g 2 h p.i.) and the bladder (6.48 ± 2.18%ID/g 1 h p.i. and 11.7 ± 2.51%ID/g 2 h p.i.) (Fig. 8). No significant increase in bone uptake was observed between 1 and 2 h p.i. (Table 2). The LNCaP tumor showed radiotracer uptake of 9.11%ID/g.

**Table 2:** Results of the ex vivo biodistribution study of ^18^F-PSMA-11. Data is reported as mean ± SD. Data for ^68^Ga-PSMA-11 was adapted from Lütje et al.^36^. *p.i.* post injection.

	^68^Ga-PSMA-11 Lütje et al.		^18^F-PSMA-11		p-value
	1 h p.i.	2 h p.i.	1 h p.i.	2 h p.i.	
	Mean ± SD	Mean ± SD	Mean ± SE	Mean ± SE	
Blood	0.4 ± 0.4	0.3 ± 0.2	0.75 ± 0.62	0.47 ± 0.07	
Bone_femur			1.26 ± 0.71	1.86 ± 0.32	1
Bone_radius/ulna			1.81 ± 0.89	1.96 ± 0.27	1
Bone_sternum			1.14 ± 0.25	1.80 ± 0.35	0.24
Bone_skull			1.76 ± 0.36	1.94 ± 0.42	1
Bone (mean)	0.1 ± 0.0	0.1 ± 0.0	1.49 ± 0.62	1.88 ± 0.32	
Bone marrow	0.7 ± 0.6	0.2 ± 0.1			
Kidneys	101.0 ± 8.8	105.8 ± 13.8	94.0 ± 27.19	82.5 ± 21.75	
Bladder			6.48 ± 4.36	11.7 ± 5.02	
Liver	0.4 ± 0.2	0.3 ± 0.0	0.39 ± 0.16	0.40 ± 0.17	
Tumor	10.4 ± 2.3	7.9 ± 1.3	9.11		

## Discussion

^18^F-PSMA-11 is a recently developed, ^18^F-labeled PSMA radiotracer. It is composed of the same Glu-urea-Lys pharmacophore and HBED-CC chelator as the widely evaluated ^68^Ga-PSMA-11. The advantageous physical properties of fluorine-18 could lead to improved visualization and delineation of tumors, especially for small lesions. ^18^F-PSMA-11 has already been evaluated in a Phase I and Phase II clinical trial in our hospital. The Phase II study was set up in order to determine an optimized scan protocol. Although several parameters such as dosage, scan duration and time of imaging post radiotracer administration were investigated, the latter was limited to two timepoints (early (1 h p.i.) and delayed (3 h p.i.) imaging) due to practical considerations inherent to a clinical trial involving human participants^14^. In vitro and in vivo evaluation of ^18^F-PSMA-11 involving dynamic imaging in mice may provide more insight into the affinity, scan time window and biological behavior of the radiotracer.

In vitro characterization of ^18^F-PSMA-11 and ^68^Ga-PSMA-11 revealed a high affinity for LNCaP cells (K_d_ value of 2.95 ± 1.50 nM and 0.49 ± 0.20 nM, respectively). Similar K_d_ values were determined for ^18^F-PSMA-11 by Malik et al*.* (10.3 ± 2.2 nM in C4-2 cells)^10^ and for ^68^Ga-PSMA-11 by Wang et al*.* (4.3 ± 0.8 nM in LNCaP cells)^19^ and Sanchez-Crespo et al*.* (27.05 nM in LNCaP cells)^20^. The recently evaluated ^18^F-PSMA-BCH demonstrated a comparable K_d_ value of 2.90 ± 0.83 nM in 22Rv1 cells^21^.

A significantly higher uptake in LNCaP compared to PC-3 xenografts indicated high specificity of PSMA-targeting radiotracers for PSMA-positive tumors. Due to the poor-differentiated and highly aggressive character of PC-3 cells, ^18^F-FDG uptake was expected to be higher compared to LNCaP cells^22–24^. However, a significant difference could only be observed in SUV_max_.

All mice underwent dynamic imaging for 2.5 h to evaluate the optimal scan window and to assess the feasibility of delayed imaging with either ^68^Ga or ^18^F as radio-isotope. The SUV_mean_ and SUV_max_ values of ^18^F-PSMA-11 suggest a wide scan time window as no significant difference was found between early (60 min p.i.) and delayed (150 min p.i.) imaging. TBR_mean_ and TBR_max_ values continued to rise up to 150 min p.i. This can be attributed to decreasing background activity due to fast radiotracer clearance. These preclinical results suggest an optimal scan time window between 1 and 2 h p.i. to obtain the highest SUV_mean_ and SUV_max_ values. Rising TBR_mean_ and TBR_max_ values at later timepoints could potentially be beneficial for suspicious lesions that were unclear on early images. This corresponds with the results obtained in the Phase II study, which suggested early imaging at 1 h p.i.^14^. Based on the preclinical data, the scan time could potentially be extended to up to 2 h p.i. A preclinical study by Cardinale et al.^25^ evaluating one LNCaP xenograft bearing mouse after administration of 25 MBq ^18^F-PSMA-1007 revealed an SUV_mean_ of approximately 1.1 in the tumor 10 min p.i., which remained constant up to 1 h and showed limited bone uptake (SUV_mean_ of approximately 1) which was reduced by half over time. The tumor was visible 20–40 min p.i. and displayed increasing image contrast over time. Comparable results were reported for ^18^F-DCFPyL where five mice were injected with 2–10 MBq and underwent dynamic PET imaging for 60 min. SUV_mean_ values reached a maximum 10 min p.i. and remained constant over time (1.1 ± 0.1 at 60 min p.i.)^26^.

Similar trends regarding tumor uptake in function of time were found for ^68^Ga-PSMA-11, although the curves for all uptake parameter were considerably lower in comparison with those for ^18^F-PSMA-11. SUV_mean_ reached its maximum value at 20 min p.i. and remained constant over time (1.05 ± 0.07 20 min p.i. to 0.97 ± 0.12 150 min p.i.).

Both for early (60 min p.i.) and delayed (150 min p.i.) acquisition, uptake parameters were significantly higher for ^18^F-PSMA-11 compared to ^68^Ga-PSMA-11 (except for TBR_max_ and SUV_max_ 150 min p.i.). Results on early vs delayed imaging (Fig. 4) suggest improved imaging with ^68^Ga-PSMA-11 at later timepoints as SUV_max_, TBR_mean_ and TBR_max_ were significantly higher at 150 min p.i. Delayed imaging using ^68^Ga-PSMA-11 seems to be favorable and may provide improved tumor visualization compared to early imaging, while limited additional benefits could be found for ^18^F-PSMA-11 imaging at later timepoints. A comparable conclusion was reached in the Phase II clinical study where no additional lesions were found between 1 and 3 h p.i. for ^18^F-PSMA-11^14^. Several clinical trials have evaluated delayed imaging with ^68^Ga-labeled PSMA-targeting radiotracers such as ^68^Ga-PSMA-11 and ^68^Ga-PSMA-I&T. A study by Afshar-Oromieh et al.^27^ reported higher lesion uptake and contrast at 3 h p.i. which lead to an increased detection rate. Schmuck et al.^28^ confirmed improved lesion contrast, but only found a limited impact on detection rates due to higher image noise and low residual activity 3 h p.i. Rahbar et al.^29^ and Derlin et al.^30^ found no additional benefit to delayed imaging with ^68^Ga-PSMA-11 because of high and variable urinary activity. However, combined with the administration of a diuretic, it could be beneficial for unclear lesions on early images and for improved assessment of the prostate gland/bed and pelvic lymph nodes. Since these studies do not report an unambiguous result, there is a need for further clinical research regarding the benefits of delayed imaging.

Even though increasing TBR values seem to be in favor of delayed acquisition, early imaging as soon as 20 min p.i. was shown to be feasible by Behesti et al*.*^31^, which would be beneficial in clinical practice due to the short half-life of ^68^Ga.

Qualitative comparison of PET images revealed improved tumor visualization and delineation with ^18^F-PSMA-11. This can be attributed to the lower positron energy of ^18^F (0.65 vs 1.90 meV) resulting in a shorter positron range (*R*_max_ 2.4 mm vs 9.2 mm), as well as the higher positron yield (97% vs 89%), which both contribute to a better image spatial resolution^9,32^. These observed differences will likely be less significant in clinical practice due to the difference in spatial resolution between preclinical (1.3 mm) and clinical PET cameras (4.5 mm), as the resolution is the limiting factor instead of isotope ranges^33^. This will be further investigated in a Phase 3 clinical trial (ClinicalTrials.gov identifier NCT03911310).

Ex vivo biodistribution of ^18^F-PSMA-11 in healthy mice demonstrated a high %ID/g in the kidneys and bladder, which can be attributed to both renal clearance of the radiotracer as well as specific binding due to PSMA expression in mouse kidneys^34^. Lütje et al*.* reported lower ^18^F-PSMA-11 uptake in the kidneys (36.7 ± 9.3%ID/g vs 94.2 ± 13.6%ID/g 1 h p.i. and 43.5 ± 5.7%ID/g vs 82.5 ± 10.8%ID/g 2 h p.i.)^35^. They also reported higher renal accumulation of ^68^Ga-PSMA-11 which was in agreement with high SUV_mean_ values in the bladder (Fig. 6). ^18^F-PSMA-11 could therefore be more suitable for the detection of lesions in the proximity of the bladder although administration of sufficient fluids, co-administration of a diuretic and voiding prior to imaging may be sufficient to decrease activity in the urinary system. Low and constant liver values of 0.40%ID/g both at 1 h and 2 h p.i. as well as rapidly decreasing SUV_mean_ values confirmed limited hepatobiliary clearance, which is advantageous for the detection of prostate cancer lesions in the pelvic region and/or abdominal cavity and potential liver metastasis^1^.

Potential defluorination of ^18^F-labeled PSMA tracers is of great concern because free ^18^F could lead to aspecific bone uptake, causing the detection of false positive lesions. Therefore, bone uptake was evaluated by in vivo PET imaging and ex vivo biodistribution. SUV_mean_ values of the spine, femur, sternum and humerus showed decreasing time activity curves up to 30 min p.i., corresponding to tracer distribution in the blood, followed by a limited rise of SUV_mean_ between 60 and 150 min p.i., although this increase was not significant. Ex vivo biodistribution showed similar results, no significant increase in bone uptake was found between 1 and 2 h p.i. The highest uptake in bone was seen 2 h p.i. in the humerus (1.96%ID/g) and skull (1.94%ID/g), which is considerably lower than tumor uptake (9.11%ID/g). Bone uptake was also lower compared to previously published results. Lütje et al*.*^35^ reported bone uptake of 3.3 ± 0.6 and 5.0 ± 0.6%ID/g at 1 h and 2 h p.i. They administered additionally 10% free ^18^F-AlF together with ^18^F-PSMA-11, which evidently led to increased bone activity (7.1 ± 1.3%ID/g and 7.0 ± 0.8%ID/g at 1 h and 2 h p.i.) but did not cause interference on the visualization of subcutaneous xenograft tumors. A comparative study between ^68^Ga-PSMA-11, ^18^F-PSMA-1007 and ^18^F-PSMA-11 by Ioppolo et al*.*^36^ reported bone uptake of 1.5 ± 0.3%ID/g and 0.9 ± 0.1%ID/g 4 h p.i. for ^68^Ga-PSMA-11 (n = 3) and ^18^F-PSMA-1007 (n = 3) compared to 4.0 and 10.2%ID/g 1 h and 4 h p.i. for ^18^F-PSMA-11 (n = 2)^36^, which was explained by rapid degradation due to instability of the HBED-CC and ^18^F-AlF complex. Although there are contradicting results regarding stability of ^18^F-PSMA-11 in serum^10,11,37,38^, ex vivo biodistribution results and PET images in this study as well as in the clinical trials did not suggest extensive tracer degradation, as the Phase 1 study showed only limited amounts of free fluoride in blood over time (increase of 1.4% and 2.5% at 50 versus 20 min p.i. and 90 versus 50 min p.i., respectively)^13^. Evaluation of possible interference of free ^18^F on bone lesion visualization should be further investigated in a preclinical bone metastasis model.

A limitation of this study was the difference in specific activity between the two PSMA-11 radiotracers. The specific activity of ^18^F-PSMA-11 was set on 20 MBq/µg as this was practically achievable due to the longer half-life and the semi-automated production method, while the short half-life of ^68^Ga and limited yield of a ^68^Ge/^68^Ga generator, especially at the end of its life cycle, only allowed lower specific activities of 1.5 MBq/µg. However, the difference in SA reflects a major advantage of ^18^F-labeled radiotracers in clinical practice. Two mice were scanned per day and equal specific activities per radiotracer were aimed for.

## Conclusion

This paper evaluated the intra-individual comparison of ^18^F-PSMA-11 and ^68^Ga-PSMA-11 for imaging of PSMA positive tumors. Both radiotracers showed high affinity for the PSMA receptor. All uptake parameters (except for SUV_max_ and TBR_max_ at 150 min p.i.) were significantly higher for ^18^F-PSMA-11 compared to ^68^Ga-PSMA-11. Delayed acquisition imaging with the latter may improve lesion detection compared to early imaging, while no additional benefits could be found for late ^18^F-PSMA-11 imaging. No significant increase in bone uptake could be found. In the preclinical setting, ^18^F-PSMA-11 demonstrated excellent imaging characteristics. Whether these can be translated to a clinical setting, will be further investigated in a Phase 3 clinical trial.

## Supplementary Information

Supplementary Figure S1.
