# Supplementary material for: Intra-individual dynamic comparison of 18F-PSMA-11 and 68Ga-PSMA-11 in LNCaP xenograft bearing mice
Source: Sci Rep. 2020 Dec 3;10:21068. doi: 10.1038/s41598-020-78273-7 (PMC7713063; doi:10.1038/s41598-020-78273-7)
Supplement: Supplementary file 1 — Supplementary Figure S1. [file 41598_2020_78273_MOESM1_ESM.docx]

Supplemental data

**Intra-individual dynamic comparison of ^18^F-PSMA-11 and ^68^Ga-PSMA-11 in LNCaP xenograft bearing mice**

Sarah Piron*^1^, Jeroen Verhoeven^1^, Benedicte Descamps^2^, Ken Kersemans^3^, Kathia De Man^3^, Nick Van Laeken^3^, Leen Pieters^4^, Anne Vral^4^, Christian Vanhove^2^, Filip De Vos^1^

^1^ Laboratory for Radiopharmacy, Ghent University, Ghent, Belgium

^2^ IBiTech-MEDISIP, Department of Electronics and Information Systems, Ghent University, Ghent, Belgium

^3^ Dept Medical Imaging, Ghent University Hospital, Ghent, Belgium

^4^ Department of Human Structure and Repair, Ghent University, Ghent, Belgium

S1:
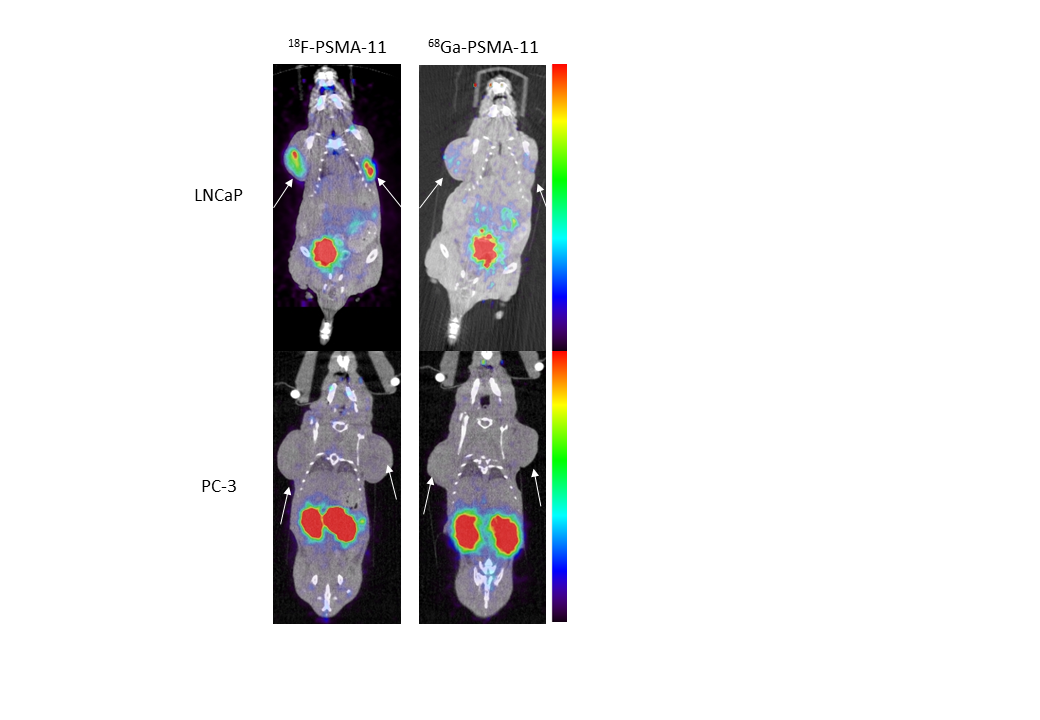
 Comparison of ^18^F-PSMA-11 and ^68^Ga-PSMA uptake with colormaps set to equal thresholds.

Comparison of ^18^F-PSMA-11 and ^68^Ga-PSMA uptake in PSMA-positive (LNCaP) and PSMA-negative (PC-3) tumors (indicated by white arrows) 1h p.i.. Colormaps were set to equal thresholds.
